# Supplementary material for: Active site specificity profiling datasets of matrix metalloproteinases (MMPs) 1, 2, 3, 7, 8, 9, 12, 13 and 14
Source: Data Brief. 2016 Feb 22;7:299–310. doi: 10.1016/j.dib.2016.02.036 (PMC4777984; doi:10.1016/j.dib.2016.02.036)
Supplement: Supplementary file 10 — Supplementary material [file mmc10.zip › WebPICS_hMMP7_T_1%/P3.html]

 

PICS results


|  |  |
| --- | --- |
| **P3\_A**  35 in 291 sites   12.0 %    effects > 10 perc. pnts.  (vice-versa in brackets)  P1\_S: 11.8 (17.2) |  |
| **P3\_E**  20 in 291 sites   6.9 %    effects > 10 perc. pnts.  (vice-versa in brackets)  P2\_Y: 11.6 (23.1) |  |
| **P3\_I**  29 in 291 sites   10.0 %    effects > 10 perc. pnts.  (vice-versa in brackets)  P3prime\_A: 14.2 (10.5) |  |
